# Matching Algorithm for 3D Point Cloud Recognition and Registration Based on Multi-Statistics Histogram Descriptors

**DOI:** 10.3390/s22020417

**Published:** 2022-01-06

**Authors:** Jinlong Li, Bingren Chen, Meng Yuan, Qian Zhao, Lin Luo, Xiaorong Gao

**Affiliations:** School of Physical Science and Technology, Southwest Jiaotong University, Chengdu 610031, China; chenbingren@my.swjtu.edu.cn (B.C.); baiguo@my.swjtu.edu.cn (M.Y.); zhaoqianddm@my.swjtu.edu.cn (Q.Z.); happyluolin@swjtu.edu.cn (L.L.); gxr@swjtu.edu.cn (X.G.)

**Keywords:** three-dimensional point cloud, feature descriptor, key point matching algorithm, 3D surface matching

## Abstract

Establishing an effective local feature descriptor and using an accurate key point matching algorithm are two crucial tasks in recognizing and registering on the 3D point cloud. Because the descriptors need to keep enough descriptive ability against the effect of noise, occlusion, and incomplete regions in the point cloud, a suitable key point matching algorithm can get more precise matched pairs. To obtain an effective descriptor, this paper proposes a Multi-Statistics Histogram Descriptor (MSHD) that combines spatial distribution and geometric attributes features. Furthermore, based on deep learning, we developed a new key point matching algorithm that could identify more corresponding point pairs than the existing methods. Our method is evaluated based on Stanford 3D dataset and four real component point cloud dataset from the train bottom. The experimental results demonstrate the superiority of MSHD because its descriptive ability and robustness to noise and mesh resolution are greater than those of carefully selected baselines (e.g., FPFH, SHOT, RoPS, and SpinImage descriptors). Importantly, it has been confirmed that the error of rotation and translation matrix is much smaller based on our key point matching algorithm, and the precise corresponding point pairs can be captured, resulting in enhanced recognition and registration for three-dimensional surface matching.

## 1. Introduction

As the laser 3D scanning technology has been developed rapidly, the recognition and registration of three-dimensional objects have also become the active and difficult problems in the research of computer vision [1]. In different kinds of 3D data description, retaining details with space-efficient data are the advantages of point cloud, which has been extensively used in 3D data processing [2]. The descriptor establishment and key point matching are two important steps of 3D surface matching. As long as the surface matched well, the accuracy of recognition and registration can be improved [3,4]. In this paper, we focus on establishing an effective feature descriptor and improving the performance of key point matching algorithm, finally resulting a satisfied 3D surface matching.

In the process of 3D surface matching, serving as a concise representation of point cloud, the descriptor is an essential component containing extensive local features. We also consider the establishment of descriptors as a feature extraction process. Due to the limitation of scanning equipment and environment, inevitably there are noise, occlusion and incomplete regions in the collected point cloud. Thus, the geometric and semantic information would be lost, which would severely affect the performance of descriptors [5]. Therefore, an effective descriptor should have a strong description ability and be robust to the noise, occlusion and incomplete regions.

In the literature on point cloud, some descriptors construct a Local Reference Frame (LRF) base on key point, and extract the spatial distribution features (e.g., the number of points) in the several partitioned bins. They have a good performance against noise and incomplete regions [4,6,7], but some of them do not have enough description ability towards point cloud with high quality. Some other descriptors extract the geometric attributes features (e.g., normals and curvatures) directly, and these descriptors have a strong description ability but they are sensitive to noise and incomplete regions in the point cloud [8,9].

Another important component is key point matching, aiming to build up a correspondence between two 3D point clouds, the most commonly used algorithms include Nearest Neighbor and Nearest Neighbor Distance Ratio [10], etc. However, they usually considered up to the top-two similar key points in the target point cloud. In fact, the correctly matched corresponding key point in the target point cloud might not be any of the top-two similar key points, which would lead to errors in the calculation of transformation matrix by Singular Value Decomposition (SVD) method [11]. Thus, the performance of 3D surface matching based on the above algorithms should be further improved.

In this paper, we mainly study the 3D surface matching problems based on the local feature descriptor of the point cloud. With the purpose of describing a 3D object from multiple aspects to enhance the description ability and robustness, we propose a Multi-statistics Histogram Descriptor (MSHD) that combines the spatial distribution and geometric attributes features. Furthermore, we propose a key point matching algorithm that not only considers more similar points when matching a key point, but also handles the corresponding key points through BP networks. Our methods perform better with higher accuracy when matching multi-object point clouds with noise, incomplete regions, and occlusion. The main contributions of this paper can be summarized as follows.

1. First, a descriptor with multi-statistical feature description histogram is proposed. A Local Reference Frame is constructed, and the normals, curvatures, and distribution density of the neighboring points are extracted; the descriptor could describes the features from these three aspects so that it keeps a strong descriptive ability and robustness to noise and mesh resolution.
2. Second, based on deep learning a new key point matching algorithm is proposed, which could detect more corresponding key point pairs than the existing methods. The experimental results show that the proposed algorithm is effective on 3D surface matching.
3. Finally, the matching algorithm based on MSHD is applied to the real component data of the train bottom. Based on this algorithm, more corresponding key point pairs in the two point clouds are obtained, resulting in a high accuracy of 3D surface matching.

The rest of this paper is organized as follows. Related work is discussed in Section 2, and Section 3 introduces the three-dimensional surface matching methods in detail, including the Multi-statistics Histogram Descriptor and matching algorithm for key point. Section 4 shows the experimental results to prove the effectiveness and feasibility of our methods. Finally, the conclusion is given in Section 5.

## 2. Related Work

In order to introduce some related work about local descriptors, feature extraction and some 3D surface matching algorithms in recognition and registration, we divide this section into two parts: feature description and extraction, and the matching algorithm of recognition and registration.

### 2.1. Feature Description and Extraction

Local feature descriptors can be divided into spatial distribution feature descriptors and geometric attributes feature descriptors, and both of them are established through the statistics of the neighboring point characteristics.

**Figure 1:**
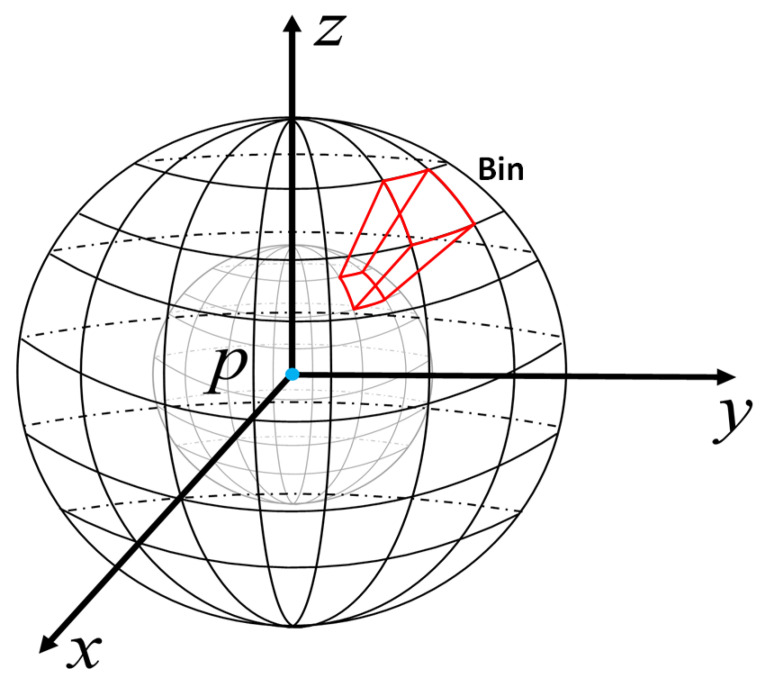
Construction of the bins in USC.

Spatial distribution feature descriptors usually construct a Local Reference Frame (LRF) based on the key point, and then divide the neighboring regions into several bins according to the LRF. Some spatial distribution measurements can be obtained in the bins, such as the number of points and density of each spatial bin. For example, as the Unique Shape Context(USC) [12] shows in Figure 1, an LRF is constructed for the key point *p*, and its spherical neighboring region can be divided into *N* bins along the radius, longitude and latitude directions, where the red volume is a bin. For each bin, there is a measurement value, and the value is sorted into a 1×N dimensional array in a certain order. The array can be regarded as a histogram, and a histogram descriptor is generated.

Spatial distribution descriptor reflects the distribution of all neighboring points. The experiments of Guo et al. [5] show that this kind of descriptors are robust to noise, occlusion, and incomplete regions, so using this kind of descriptors as the representation of the point cloud is an effective choice when the quality of the point cloud is not good. Geometric attributes feature descriptors usually calculate some geometric values, for example, the coordinates, normals, and curvatures of each neighboring point, and the descriptors represent the specific attributes of each neighboring point [5]. This geometric measurements are more complex than the number or density of the points in each bin, so a stronger description ability can be provided. For a high-quality point cloud without too much noise or occlusion, establishing the geometric attributes feature descriptor is a good choice.

There are some work reported about these two kinds of descriptors. The Spin Image (SI) algorithm is introduced to establish a cylindrical coordinate system for a local point cloud and rotate it around the axis to obtain a two-dimensional projection point cloud, and then a histogram descriptor is constructed [6]. Some work makes a further improvements on the basis of SI. Compared with SI, a global orientation is used for recognizing different types of objects [7]. The Intrinsic Spin Image (ISI) is invariant to isometric shape deformations, enjoying the high expressiveness of the nonparametric spin image descriptors [13]. The Point Feature Histograms (PFH) and Fast Point Feature Histograms (FPFH) are established in Darboux coordinate system for points in the neighborhood that meet the requirements, then the angles are counted between the normal vector and the direction vector of the coordinate axis, and finally a statistical histogram is built up [8,14]. The Local Surface Patch(LSP) algorithm includes two parts: histograms and attributes of points [15]. Sun et al. [16] perform the Laplace–Beltrami calculation on the local surface of 3D point cloud and obtains the Heat Kernel Signature (HKS) by embedding calculations into the derived space. The Signature of Histograms of Orientations (SHOT) divides the local spherical neighborhood on the radial, longitude, and latitude direction, and counts the number of points that fall into each subspace. In the subspace, the distribution of the cosine values of the angle between the normal vectors of the neighboring points and those of the center key point are calculated respectively. Finally, the histogram of the normal vector angle distribution of each subspace is spliced to form a SHOT descriptor [9]. For the Rotational Projection Statistics (RoPS) descriptor, an LRF needs to be constructed for the local area, and then the neighboring points are moved along the three coordinate axes of the LRF. Rotating and projecting for many times, counting the central moment and Shannon entropy of the projected image at the same time [4]. Bin Lu et al. [17] proposed a multi-scale feature and covariance matrix descriptor, including the geometric angle, dimension, projection length ratio, and curvature difference.

Furthermore, there are many feature extraction methods based on deep learning, such as those in references [18,19,20,21]. The PointNet uses multi-layer perceptrons to perform feature extraction on the local area of the point cloud. Through the transformation of features, the network model has the permutation invariance about the input points, and then the pooling layer is used for realizing the construction of global features so that different tasks such as classification, semantic segmentation and partial segmentation could be completed finally [18]. For the first time, point cloud is directly used as the input to realize point cloud recognition. However, PointNet directly uses all points to participate in feature extraction, but it can not extract the local features of the points, which limits the ability of recognizing the detailed patterns in a complex scenes. Therefore, Qi et al. [19] proposed PointNet++ on this basis. The point cloud is pre-partitioned to improve the ability in extracting the detailed features the better result has been achieved than PointNet does. This network model is widely used in segmentation and recognition, which indirectly explains the importance of local features. However, these methods still have some problems, such as low space efficiency or space storage, low robustness to noise and resolution, etc.

Considering that learning-based methods required large training data, it is important to establish an effective and robust feature descriptor for point cloud. We hope it not only has the advantages of being robust to noise and incomplete regions like spatial distribution feature descriptors, but also has a well-performed description ability like geometric attributes feature descriptors; in another words, it combines spatial distribution features and geometric attributes ones. Therefore, how to establish this descriptor is a challenge.

### 2.2. The Algorithm of Recognition and Registration

**Figure 2:**
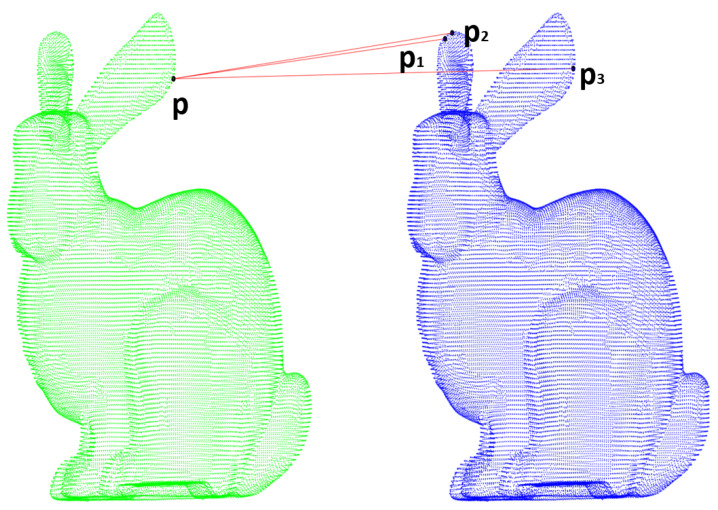
The process of key points matching in two point clouds.

3D point cloud key point matching algorithms often use the Nearest Neighbor (NN) and Nearest Neighbor Distance Ratio (NNDR) [10]. As Figure 2 shows, suppose there is a key point *p* from the origin point cloud waiting for matching to a key point in the target point cloud. $p_{1}$, $p_{2}$, and $p_{3}$ are detected as the most three similar key points through comparing their similarity of descriptors, and they are sorted according to the degree of similarity. NN directly regards the most similar points $p_{1}$ and *p* as a corresponding point pair. Depending on the ratio of similarity, NNDR chooses $p_{1}$ or $p_{2}$ as the corresponding point to p, and it only considers the top-two similar key points. Sometimes, maybe the true corresponding point is sorted lower, for example, $p_{3}$ is actually the correct corresponding key point to *p* in Figure 2. Then, a wrongly matched corresponding key point pair might be caused by these methods, and this would lead to errors in the calculation of transformation matrix by SVD.

For solving the effects from wrong corresponding key point pairs, Iterative Closest Point algorithm (ICP) is often used as a fine calibration after poor surface matching results in the registration, which has a good performance [10,22]. First, the ICP algorithm requires the entire point cloud to participate in the iteration, so the computing efficiency is low. Second, the ICP based on local iterative optimization is susceptible to the influence of local minimums, so it requires the initial position of the two matching point clouds as close as possible, the initial overlap rate as high as possible, and only a final result of local optimality is guaranteed [23]. In recent years, there have been some works concentrated on the improvement of ICP algorithm. For example, Velocity Updating ICP(VICP) solved the problem about the error during the object in movement [24]. Go-ICP has a high speed for fine registration [25]. The point-to-line ICP (PLICP) takes the distance from the point to the line as the error, and it has a faster convergence speed [26]. Furthermore, there is the 3D-NDT method which uses the probability density instead of the feature extraction and the matching of corresponding points [27]. Chang et al. [28] used the K-means clustering method to obtain the corresponding point pairs. The method proposed by Li et al. [29] can extract the overlapping area of two point clouds, which greatly improves the accuracy of registration. He et al. [30] combined PointNet++ network and the ICP algorithm for training, and the result of registration was robust with the high speed, but it has unsatisfactory performance on sparse point clouds, because they cannot provide enough features. Kamencay et al. [31] use the Scale-Invariant Feature Transform (SIFT) function for the initial alignment transformation of the point clouds, combining with the K-nearest neighbor algorithm and using the weighted ICP algorithm for registration. Aiming to solve problems such as low convergence speed due to uncertainty of initial transformation matrix and difficulty of accurate matching for corresponding points, Xiong et al. [32] proposed a novel feature descriptor based on ratio of rotational volume and an improved coarse-to-fine registration pipeline of point clouds, and experimental results show that the improved pairwise registration pipeline is effective in pairwise registration.

No matter ICP or its improved algorithms, before using them, the key of registration is to get more correct corresponding key point pairs, so a better initial position of two point clouds can be obtain. In the recognition of point cloud, the works in [33,34] use Random Sampling Consensus Algorithm (RANSAC) for eliminating wrong corresponding point pairs. RANSAC chooses three groups of corresponding point pairs to make registration and then calculates the distance of the rest corresponding point pairs after registration to evaluate the accuracy of the three groups of corresponding point pairs, so as to find the most accurate three groups of corresponding point pairs by iterations; finally, the accuracy of correspondence between two point clouds can be improved. However, RANSAC needs a large number of iterations so that the calculation efficiency is low, and changing the thresholds will totally affect the final results. In general, the matching algorithm should find more correct corresponding key point pairs in matching stage to obtain a better performed 3D surface matching results, which would enhance the accuracy of registration and recognition.

## 3. Methodology

### 3.1. Multi-Statistics Histogram Descriptors

**Figure 3:**
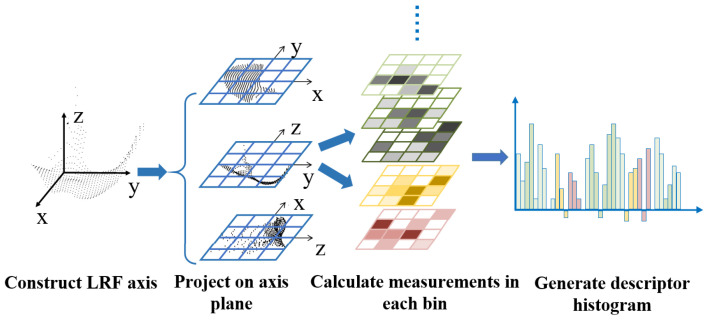
Establishment process of descriptors.

With the purpose of establishing an effective descriptor, we hope it to have both the advantages of spatial distribution feature descriptors and geometric attributes feature descriptors: being robust to noise and incomplete regions with a well-performed description ability at the same time. Therefore, in this paper we propose a descriptor with multi-statistical feature description histogram, which combines the spatial distribution and geometric attributes features. First, we construct an LRF based on the key point, and three coordinate axis planes can be obtained. All the neighboring points can be projected onto these axis planes, so we can calculate the density and average curvatures based on the points falling into each bin, and calculate the normals of the points in each bin. Meanwhile the values are sorted into a 1XN dimensional array with a certain order. The array can be regarded as a histogram, and a histogram descriptor is generated. Figure 3 shows the process of the establishment of the proposed descriptors.

#### 3.1.1. Construct an Local Reference Frame

In the point cloud, if the coordinate system changed, the coordinate of points would also change. For eliminating the influence of the coordinate system changes on the description, some related researches use the invariance of three-dimensional rigid body space transformation, for constructing an Local Reference Frame. First, all the points in the target regions are translated to the centroid, and rotated around the origin of the new coordinate system, which is constructed based on centroid, until the original axes of the target region points are parallel to the three main axes directions. This is the process of Local Reference Frame being constructed.

Supposed there is a point cloud $P=\left\{p_{1},p_{2},\ldots ,p_{i},\ldots ,p_{n}\right\}$ with n points, and any point $p_{i}$ in *P* could construct an LRF in the neighborhood of $p_{i}$. Here, the neighboring points of $p_{i}$ within a certain radius are defined as $nbhd\left(p_{i}\right)$. For eliminating the influence of the translation, the $nbhd\left(p_{i}\right)$ is translated to the coordinate system, which is constructed based on the centroid of the neighborhood:

(1) $$p_{c}=\sum_{p_{j}\in nbhd\left(p_{i}\right)}^{}\frac{p_{j}}{k}$$

(2) $$p_{j}{}^{\prime }=p_{j}-p_{c}$$

where $p_{c}$ is centroid of $p_{i}$ neighborhood, $p_{j}$ is all the coordinates of the neighboring points in the neighborhood of $p_{i}$, $p{}_{j}{}^{\prime }$ is the coordinates of the neighboring points after transforming to the centroid coordinate system, and *k* is the number of the neighboring points of $p_{i}$.

Expect the centroid $p_{c}$ point, and the key point can also be set as the origin of the new coordinate, the coordinates formula is

(3) $$p_{j}{}^{\prime }=p_{j}-p_{k}$$

where the $p_{k}$ is the coordinate of key point. Then, Principal Component Analysis (PCA) is performed to eliminate the influence of rotation. A covariance matrix $cov\left(p_{i}\right)$ is constructed for the translated $nbhd\left(p_{i}\right)$ by the following formula:

(4) $$cov\left(pi\right)=\frac{1}{k}\sum_{p_{j}\in nbhd\left(p_{i}\right)}^{}{\left(p_{j}-p_{c}\right)}^{T}\left(p_{j}-p_{c}\right)$$

If the key point is used as the origin, $p_{c}$ should be replaced with $p_{k}$ here. As $cov\left(p_{i}\right)$ is a symmetric positive semi-definite matrix, we can get three non-negative real eigenvalues $\lambda_{1},\lambda_{2},\lambda_{3}$, and they satisfy the relation of $\lambda_{1}\geq \lambda_{2}\geq \lambda_{3}$. These three eigenvalues correspond to three eigenvectors $v_{1},v_{2},v_{3}$, and build up a set of orthogonal basis. The three eigenvectors could be used as the three coordinate axes of LRF.

The process of selecting the coordinate axis should be consistent. First, the eigenvector $v_{1}$, corresponding to the largest eigenvalue $\lambda_{1}$, is chosen as the axis of x. The direction of axis z is related to eigenvector $v_{3}$, which is corresponding to the smaller eigenvalue $\lambda_{3}$, and it needs to calculate the vector component of the neighboring points along the direction of $v_{3}$. If the number of points with negative coordinates is more than that of points with positive coordinates, the direction of axis z is the same as $v_{3}$; otherwise, set axis z as the opposite direction of $v_{3}$. Now, the axis of y could be defined since axis x and axis z have been defined.

Then, the coordinates of the neighboring points after transformation can be calculated by the following formula:

(5) $$R=[v_{1},v_{3}\times v_{1},v_{3}]$$

(6) $$p{}^{\prime \prime }=p{}^{\prime }\cdot R$$

where the $p{}^{\prime }$ is the initial coordinate of the neighboring points after translation, *R* is the direction of the axis, and $p{}^{\prime \prime }$ is the coordinate of $p{}^{\prime }$ projected onto the LRF axis planes.

#### 3.1.2. Normals and Curvatures

After the LRF has been constructed, the coordinates of neighboring points could not be directly used as the measurement to generate a descriptor. The accuracy will be seriously reduced once the sampling points change or some noise invades. Therefore, the normals and the curvatures might be the better choices as the measurements to generate a descriptor. The normal of $p_{i}$ could be approximately equal to the tangent plane direction vector of the surface, which is constituted by $p_{i}$ and the neighboring points. After the covariance matrix $cov\left(p_{i}\right)$ of the $nbhd\left(p_{i}\right)$ is eigen-decomposed, the PCA algorithm also can be used for calculating the normals. The eigenvector $v_{3}$ corresponded to the smaller eigenvalue could be regarded as the direction vector of the fitting approximate plane. Therefore, $v_{3}$ represents the normal of $p_{i}$ approximately, where $n_{i}=v_{3}$. As the local surface may be concave or convex, the direction of the normals need to be clarified, and the component of the neighboring points along the $v_{3}$ direction is calculated. If the points with negative coordinates is more than those with positive coordinates, set $n_{i}=-v_{3}$. In order to make $p_{i}$ and its neighboring points distribute on the same plane approximately, the tangent plane could be replaced with an approximate plane, and the radius r of the neighborhood should not be too large. In this paper, we search the neighboring points by kNN algorithm to calculate the normals, the number of neighboring points for detecting is set as 50.

The measurements of curvature represents the steepness of the surface that constituted by the point and its neighboring points. In a word, if the curvatures of points were larger, the variation of the surface would be larger, and more features could be obtained. Otherwise the smaller curvatures that the points have, the smoother that the surface is, and fewer features could be obtained.

The curvature formula of y in two-dimensional coordinate system is as follows:

(7) $$c=\frac{y^{\prime \prime }}{{\left(1+{y^{\prime }}^{2}\right)}^{\frac{3}{2}}}$$

where y means the ordinate of the point, the curvature c is proportional to the second derivative of y, and thus the curvature is sensitive to the changes of the object surface, also it is susceptible to the interference of noise.

Based on the three eigenvalues $\lambda_{1},\lambda_{2},\lambda_{3}$ in the covariance matrix $cov\left(p_{i}\right)$, we could estimate the complexity of the surface. The curvature $c_{i}$ of $p_{i}$ can be defined as

(8) $$c=\frac{\lambda_{3}}{\lambda_{1}+\lambda_{2}+\lambda_{3}}$$

where *c* is curvature of the point, but it is an curvature approximation of the surface constituted by $nbhd\left(p_{i}\right)$.

#### 3.1.3. Generate the Descriptors

**Figure 4:**
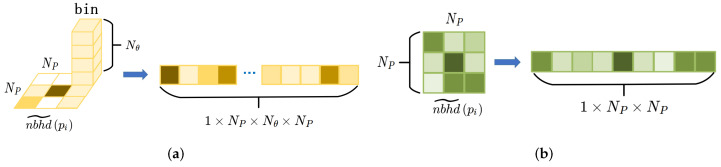
Generation of the histogram. (**a**) Grids and bins are expanded to an array. (**b**) Grids are expanded to an array.

The specific process of the descriptor generation is as follows:

(1) Preparation: Detecting the key points of the point cloud *P*, the key points are denoted as KP (the curvature *c* and the normal *n* of each point are calculated in the point cloud *P*).

(2) Construction of LRF: Searching the neighborhood of the key point $p_{i}\in KP$ in the point cloud *P*, then an LRF based on the key point $p_{i}$ can be constructed, and the coordinates of $nbhd\left(p_{i}\right)$ can be translated into LRF.

(3) Projection along the axis of LRF: The $nbhd\left(p_{i}\right)$ is projected along the three LRF coordinate axes, respectively, three frames of the projected point cloud can be obtained.

(4) Generation of the grid statistical map about the projected point cloud: Dividing the projected point cloud into $N_{P}\times N_{P}$ grids, the points and their coordinates could be obtained in each grid, and thus a discrete projection statistical map $\tilde{nbhd}\left(p_{i}\right)$ could be obtained.

(5) Construction of the normal histogram: $n_{j}$ is defined as the normals of neighboring points, and $n_{i}$ is the normal of center point $p_{i}$. The angle $\langle n_{i},n_{j}\rangle$ between $n_{i}$ and $n_{j}$ can be calculated. Then the value range of $\langle n_{i},n_{j}\rangle$ with $\left[0,\pi \right]$ can be divided into $N_{\theta }$ subintervals, and the points distributed in each subinterval of the grid can be counted. As Figure 4a shows, each sub-interval of the grid can be regarded as a bin, $N_{\theta }$ bins in each grid. With one measurement in each bin, and there is $N_{\theta }$ bins in each grid. The gird map are expanded to a $1\times N_{\theta }\times N_{P}\times N_{P}$ dimensional array in a certain order, and after normalization, the histogram $H_{n}$ is generated.

(6) Construction of the curvature histogram: Calculating the average curvature of each grid in the projected statistical map $\tilde{nbhd}\left(p_{i}\right)$, the values with the average curvature are assigned to each grid. Value 1 is assigned for the grid with no points. With one measurement in each grid, as the Figure 4b shows, the gird map are expanded to a $1\times N_{P}\times N_{P}$ dimensional array in a certain order, and after normalization, the histogram $H_{c}$ is generated.

(7) Construction of the average density of the points histogram: Calculating the average density of the points in each grid in the projected statistical map $\tilde{nbhd}\left(p_{i}\right)$, the average density values of the points are assigned to each grid, and value 1 is assigned for the grid with no points. The gird map are expanded to a $1\times N_{P}\times N_{P}$ dimensional array in a certain order, and after normalization, the histogram $H_{d}$ is generated.

(8) Splicing the feature histogram: The arrays of $H_{n}$, $H_{c}$ and $H_{d}$ from three frames can be spliced in together, so the descriptor histogram can be generated as follows:

(9) $$H=\left[k_{1}H_{n},k_{2}H_{c},k_{3}H_{d}\right]$$

where *H* is the final feature histogram descriptor, $k_{1},k_{2}$ and $k_{3}$ are weights that have been presented for adjusting the proportion of the normal, curvature and density in the feature description.

In order to evaluate the descriptor conveniently and get the values of $k_{1},k_{2}$ and $k_{3}$, we make $H_{n},H_{c}$, and $H_{d}$ the same proportions temporarily in the description.

### 3.2. Matching Algorithm

In the stage of key points matching, the key points in the model point cloud would directly match the most similar ones in the scene point cloud with NN algorithm. While the NNDR algorithm only considered the top-two similar key points in the target point cloud. In fact, the correct matching key point in the target point cloud may be not any of them, leading to errors in the calculation of transformation matrix. Both of the two matching algorithms would cause the wrong corresponding key point pairs, especially in the point cloud with low quality. Therefore, for getting more precise corresponding key points pairs effectively, we proposed a novel key point matching algorithm that not only considers more similar points, but also handles the corresponding key points through BP networks. This algorithm is divided into two parts:

(1) In the first part, suppose there are *i* key points in the model point cloud and *j* key points in the scene point cloud. We use the proposed descriptor in this paper to extract the features one by one from the key points $KP_{m}^{i},\left(i=1,2,3,\ldots ,i\right)$, and the same operation is also carried out on the key points $KP_{s}^{j},\left(j=1,2,3,\ldots ,j\right)$ in the scene point cloud. We choose a key point $KP_{m}^{i}$ in the model randomly, and set the number of similar points to be found as *k*. Therefore, key points as $KP_{s}^{i,k}$, where *i* means the key point from the scene matching $KP_{m}^{i}$. $KP_{s}^{i,1}$ means the first similar key point, and $KP_{s}^{i,2}$ means the second similar key point, etc. Defining $df\left\{P_{1},P_{2}\right\}$ as the similarity of features descriptors between these two points $P_{1}$ and $P_{2}$, calculated by kNN methods. Now, consider the following formula:

(10) $$df\left\{KP_{m}^{i},KP_{s}^{i,1}\right\}<0.5*\frac{df\left\{KP_{m}^{i},KP_{s}^{i,2}\right\}+df\left\{KP_{m}^{i},KP_{s}^{i,3}\right\}+\ldots +df\left\{KP_{m}^{i},KP_{s}^{i,k}\right\}}{k-1}$$

where $KP_{m}^{i}$ and $KP_{s}^{i,1}$ are a pair of corresponding key points. If the most similar point $KP_{s}^{i,1}$ does not satisfy the above equation, we take all the *k* similar points $KP_{s}^{i,1},KP_{s}^{i,2},\ldots ,KP_{s}^{i,k}$ into the second part to consider which key point is matched with $KP_{m}^{i}$ precisely.

**Figure 5:**
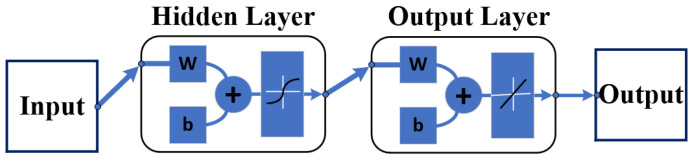
Structure of a conventional BP neural network.

(2) In the second part, we handle the corresponding key points with BP networks. The reasons of using BP network is that it could fit the mapping relationship between the independent variables $x_{1},x_{2},\ldots ,x_{n}$ and the dependent variable *y* through enough data training. The structure of a conventional BP neural network is shown in Figure 5.

The number of neurons in the hidden layer can be set based on experience as follows:

(11) $$nl=\sqrt{n+m}+b$$

where nl is the number of neurons in the hidden layer, n is the number of neurons in the input layer, m is the number of neurons in the output layer, and b is a constant within $\left[0,10\right]$.

In general, the transfer function of the hidden layer adopts the Sigmoid function, so that the BP network could achieve arbitrary approximation to any function, while the output layer adopts a linear function. For the choice of learning rate, a learning rate that is too large will lead to ups and downs in network training; also, it will easily skip the global optimal solution and enter the local optimal solution. There have been many methods in designing the learning function, and the Levenberg–Marquardt Backpropagation learning algorithm is more commonly used with a good performance and high training speed.

**Figure 6:**
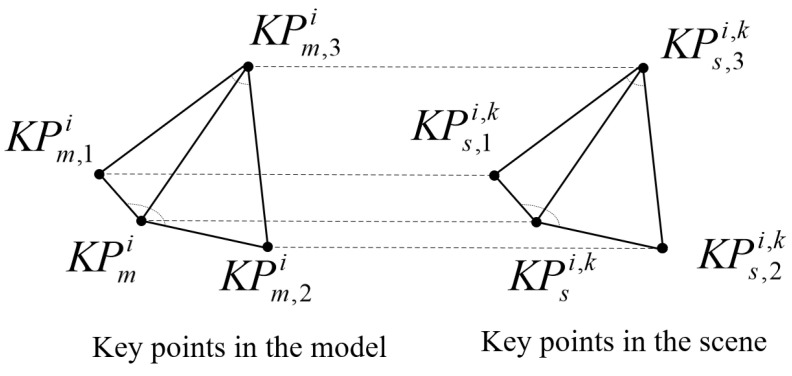
Spatial features of the corresponding key points in the model and scene point cloud.

We can calculate some spatial features such as the distance between two key points and the angles formed by three key points. These spatial features and angles can be used as the input independent variables $x_{1},x_{2},\ldots ,x_{n}$ to BP networks for training. Here, $dist\left\{P_{1},P_{2}\right\}$ is defined as the Euclidean distance difference between two points, $angle\left\{P_{1},P_{2},P_{3}\right\}$ is defined as the angle of three points with $P_{2}$ as the vertex between $P_{1}$ and $P_{3}$. As Figure 6 shows, suppose there is a key point $KP_{m}^{i}$ in the model point cloud and three nearest neighboring key points of it; they are $KP_{m,1}^{i},KP_{m,2}^{i},KP_{m,3}^{i}$. Furthermore, suppose we have found *k* similar key points to $KP_{m}^{i}$ in the scene point cloud, one of which is $KP_{s}^{i,k}$, and the three nearest neighboring key points of $KP_{s}^{i,k}$ are $KP_{s,1}^{i,k},KP_{s,2}^{i,k}$ and $KP_{s,3}^{i,k}$. Then, we can calculate the spatial distance features from $KP_{m}^{i}$ and $KP_{s}^{i,k}$ as follows:

(12) $$d_{1}=\left|dist\left\{KP_{m}^{i},KP_{m,1}^{i}\right\}-dist\left\{KP_{s}^{i,k},KP_{s,1}^{i,k}\right\}\right|$$

(13) $$d_{2}=\left|dist\left\{KP_{m}^{i},KP_{m,2}^{i}\right\}-dist\left\{KP_{s}^{i,k},KP_{s,2}^{i,k}\right\}\right|$$

(14) $$d_{3}=\left|dist\left\{KP_{m}^{i},KP_{m,3}^{i}\right\}-dist\left\{KP_{s}^{i,k},KP_{s,3}^{i,k}\right\}\right|$$

and the spatial angle features from $KP_{m}^{i}$ and $KP_{s}^{i,k}$ as follows:

(15) $$\theta_{1}=\left|angle\left\{KP_{m,1}^{i},KP_{m}^{i},KP_{m,2}^{i}\right\}-angle\left\{KP_{s,1}^{i,k},KP_{s}^{i,k},KP_{s,2}^{i,k}\right\}\right|$$

(16) $$\theta_{2}=\left|angle\left\{KP_{m,1}^{i},KP_{m,3}^{i},KP_{m,2}^{i}\right\}-angle\left\{KP_{s,1}^{i,k},KP_{s,3}^{i,k},KP_{s,2}^{i,k}\right\}\right|$$

as well as the differences of descriptors from $KP_{m}^{i}$ and $KP_{s}^{i,k}$:

(17) $$df_{1}=\left|df\left\{KP_{m}^{i},KP_{m,1}^{i}\right\}-df\left\{KP_{s}^{i,k},KP_{s,1}^{i,k}\right\}\right|$$

(18) $$df_{2}=\left|df\left\{KP_{m}^{i},KP_{m,2}^{i}\right\}-df\left\{KP_{s}^{i,k},KP_{s,2}^{i,k}\right\}\right|$$

(19) $$df_{3}=\left|df\left\{KP_{m}^{i},KP_{m,3}^{i}\right\}-df\left\{KP_{s}^{i,k},KP_{s,3}^{i,k}\right\}\right|$$

As long as there are enough precisely matched corresponding key point pairs and wrong matched corresponding key point pairs, we can get enough independent variables $d_{1},d_{2},d_{3},\theta_{1},\theta_{2}$ and $df_{1},df_{2},df_{3}$. Then, we could use these independent variables as the input data to BP networks. The label of the precise corresponding key point pairs is 1, and that of the wrong matched key point pairs is 0. Therefore, we hope the BP networks can predict a value of the input data, whether the two key points represent the corresponding key point pairs or not.

We trained the BPnet1 by using $d_{1},d_{2},d_{3},\theta_{1},\theta_{2}$ as the input data, and defined the output variable as y. We trained BPnet2 by using $df_{1},df_{2},df_{3}$, and defined the output variable as v. After trained with a huge number of data, these two BP networks performed well in validation. We combined them with the second part to judge whether the two key points are the corresponding pairs.

Defining two thresholds $\tau_{1}$ and $\tau_{2}$, and suppose we have calculated the $d_{1},d_{2},d_{3},\theta_{1},\theta_{2}$ from $KP_{m}^{i}$, $KP_{s}^{i,k}$ and their neighboring key points. Then, we input $d_{1},d_{2},d_{3},\theta_{1},\theta_{2}$ into BPnet1. If the output is $y>\tau_{1}$, we consider $KP_{m}^{i}$, $KP_{s}^{i,k}$ as a corresponding key point pair. Otherwise, we calculate $df_{1},df_{2},df_{3}$ from $KP_{m}^{i}$, $KP_{s}^{i,k}$ and their neighboring key points. Taking $df_{1},df_{2},df_{3}$ into BPnet2, if the output $v>\tau_{2}$, we can also consider $KP_{m}^{i}$, $KP_{s}^{i,k}$ as a corresponding key point pair. If the output $v<\tau_{2}$, let k=k+1, and we judge the next similar key point from the scene point cloud. If all the *k* similar key points $KP_{s}^{i,k}$ to $KP_{m}^{i}$ are not the corresponding key points, let i=i+1, and we continue to consider the next key point $KP_{m}^{i}$ in the model point cloud, whether there is a corresponding key points in or not in the scene point cloud. Finally, the corresponding key point pairs can be obtained. Here setting the threshold $\tau_{1}=0.95$ and threshold $\tau_{2}=0.7$. The BP networks would have a best performance according to the experience of validation, and surely they can be adjusted according to the specific data.

## 4. Experimental Results

### 4.1. Multi-Statistics Histogram Descriptor

#### 4.1.1. Data and Testing Environment

**Figure 7:**
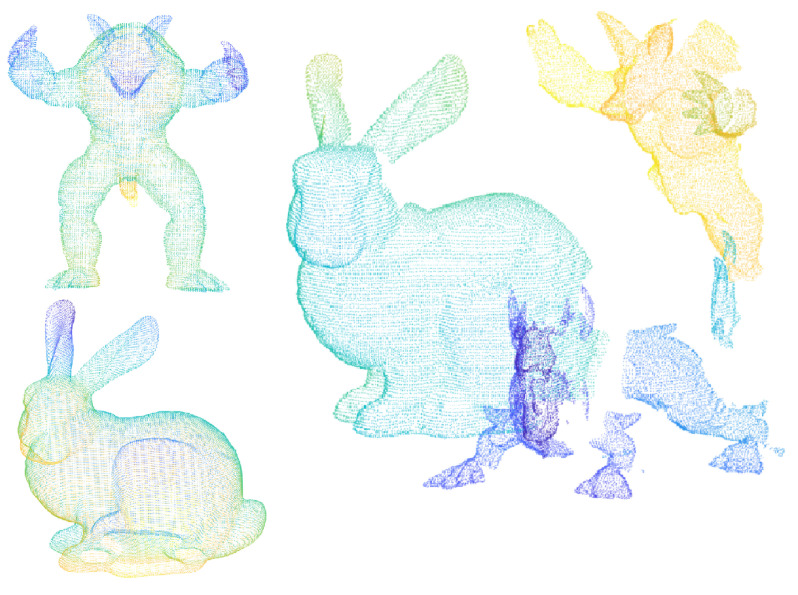
Part of the point cloud in Random Views.

There are six different models and thirty-six scenes in the dataset of Random Views, which is established on the basis of Stanford 3D dataset, and as shown in Figure 7 [5], there are some occlusion and incomplete regions in the scenes. The models are generated by registration on the multi-view point clouds, and they are from Stanford University point cloud library, including the famous Armadillo, Bunny, Happy Buddha, Asian Dragon, Thai Statue, etc. Because the mesh resolution of the laser scanning that scanned these dataset is the same, so each point cloud is scaled to the same size, and it is convenient to set the neighborhood radius *r* for the descriptor. All experiments are performed under windows10 operating system, Intel i5-9400 and 16 GB RAM with the simulation software.

As Figure 7 shows, the quality of the model point cloud is really high. In contrast, the scenes are single-view point cloud with occlusion and noise, and each scene includes three to five models, but the quality of the scene point cloud is quite low.

#### 4.1.2. Evaluation Criteria of the Descriptor

The Precision and Recall curve (P−R curve) is often used to evaluating the description ability of the local feature descriptors. The process of evaluating the descriptors are as following shows.

First of all, the key points are detected by the Intrinsic Shape Signatures (ISS) algorithm [35] in the model and the scene point cloud, and the ISS is commonly used in key point detection. Then, the descriptors are generated for each key point from the model and scene point cloud, and the feature sets $F_{model}$ and $F_{scene}$ can be obtained.

Next, the key point matching algorithm NNDR would be used for feature matching. In brief, the most similar descriptor $f_{scene}^{i}$ and the second similar descriptor $f_{scene}^{ii}$ in the scenes would be detected for each descriptor $f_{model}^{i}$ in the models. The ratio of the distance can be calculated as follows:

(20) $$\tau =\frac{|f_{scene}^{i}-f_{model}^{i}|}{|f_{scene}^{ii}-f_{model}^{i}|}$$

It can also be understood as the ratio of the similarity between $f_{model}^{i}$ with $f_{scene}^{i}$ and $f_{scene}^{ii}$.

Only if the ratio τ is less than the threshold $\tau_{th}$, and the descriptor $f_{model}^{i}$ and descriptor $f_{scene}^{i}$ are matched, the key points of these two descriptors are a corresponding key point pair. After that, by using SVD method, the transformation matrix is calculated through the corresponding key point pairs between the model point cloud and the scene point cloud. Finally, the model point cloud can be transformed to the scene point cloud.

Ideally, all the corresponding key point pairs completely overlap point to point. Due to the limitation of NNDR algorithm and the difference between the description ability of the descriptors, some wrong matched corresponding key point pairs would be caused. It should be pointed out that the wrong matched corresponding key point pairs would take some errors when calculating the transformation matrix, leading to the distance after transformation between the key point from model with the key point from scene. Therefore, we can use the same key point matching algorithm NNDR to evaluate the description ability of different kinds of descriptors.

After transformation, if the distance between two correctly matched corresponding key points is less than 0.5 r, these two corresponding key points will be regarded as the true positive correspondence; otherwise, they will be regarded as the false positive correspondence. Moreover, if the distance of wrong matched corresponding key points is more than 0.5 r, these two corresponding key points will be regarded as the false negative correspondence.

Many groups of precision and recall can be obtained by changing the ratio threshold $\tau_{th}$ in NNDR, so the P−R curve can be generated as follows:

(21) $$precision=\frac{TP}{TP+FP}$$

(22) $$recall=\frac{TP}{TP+FN}$$

where TP is the number of true positive correspondences, FP is the number of the false positive correspondences, and FN is the number of false negative correspondences.

According to the principle of NNDR, more corresponding key point pairs will be obtained when the threshold $\tau_{th}$ is raised, but the precision will be decreased, and more true positive correspondences will also be obtained, so the recall will be increased. By contrast, fewer corresponding key point pairs will be obtained due to the threshold $\tau_{th}$ is reduced, while the precision will be increased, and some correctly matched corresponding key point pairs can not be obtained, so the recall will be decreased. Thus, the P−R curve should be a decreasing curve. In general, if the precision remains high when the recall increasing, it is an effective descriptor.

#### 4.1.3. Robustness to Noise

**Figure 8:**
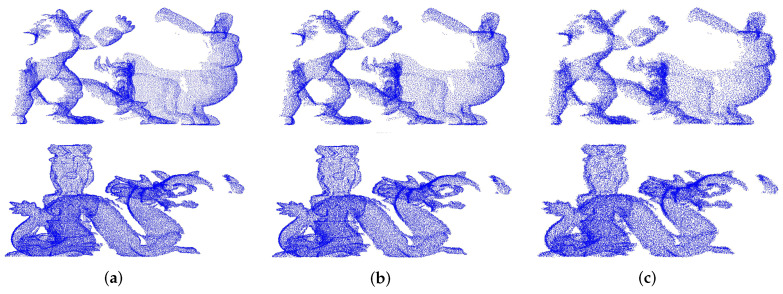
Examples of the scenes with different peak intensity of Gaussian noise. (**a**) 0.05 r. (**b**) 0.1 r. (**c**) 0.2 r.

For evaluating the robustness of the descriptor to noise, the Gaussian noise is added, respectively, with the peak intensity of 0.05 r, 0.1 r, and 0.2 r to the scene point cloud. Then, the feature descriptors based on the key points are calculated in the scenes. The feature descriptors of the model point cloud without noise are also calculated. The key point matching experiment are made for generating the P−R curve, our descriptor would be contrasted with FPFH, RoPS, SHOT, and SpinImage. Here, with different peak intensity Gaussian noise, two examples of the scene point clouds are shown in Figure 8.

The steps of the experiments are as follows.

(1) First, the key points from the model and the scene point cloud are detected and, respectively, recorded as $KP_{m}$ and $KP_{s}$. The feature descriptors are generated based on $KP_{m}$ and $KP_{s}$. The feature set $F_{model}$ is built up by all the model descriptors, and the scene feature set $F_{scene}$ is built up by all the scene descriptors.

(2) Based on the $F_{scene}$, a KD tree can be constructed. Through the kNN searching algorithm, each descriptor in $F_{model}$ can detect several similar descriptors in $F_{scene}$.

(3) Finally, the correspondences between $KP_{m}$ and $KP_{s}$ can be constructed by using NNDR. As it was mentioned in Section 4.1.2, many groups of precision and recall can be obtained by changing the ratio threshold $\tau_{th}$ in NNDR, so the P−R curve can be generated.

**Figure 9:**
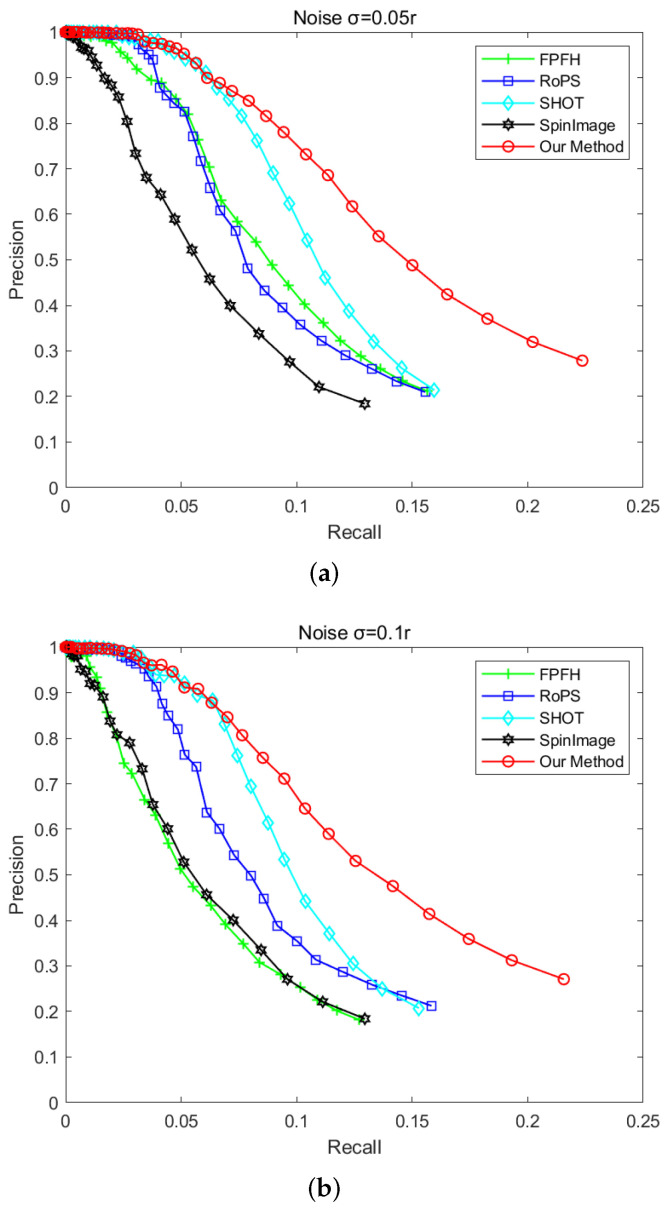

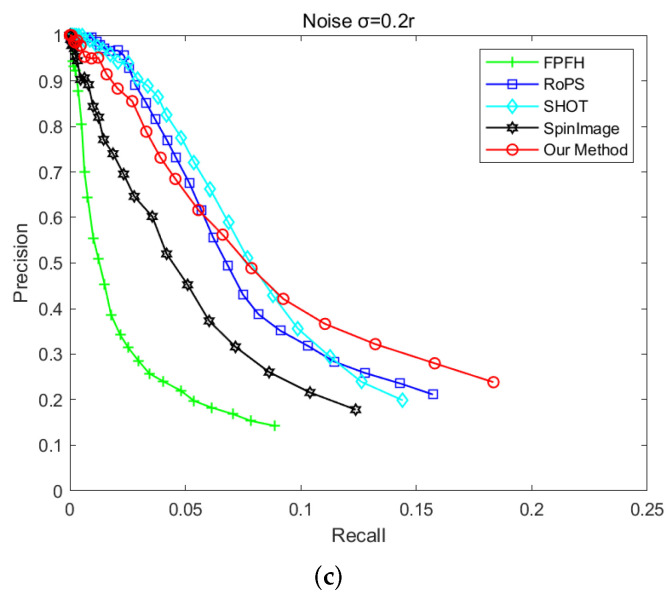
P−R curves in different noise scenes. (**a**) Gaussian noise σ = 0.05 r. (**b**) Gaussian noise σ = 0.1 r. (**c**) Gaussian noise σ = 0.2 r.

The P−R curve in Figure 9 shows the performance of these different descriptors. It can be seen that our descriptor is more robust to noise than other descriptors, and SHOT has the second best performance. This occurs because the proposed descriptor extracts the features from multiple aspects especially from the density and generates a statistical histogram. After projection, the histogram generated by the local point density is not sensitive to noise, so the robustness and description ability of descriptor is guaranteed. However, it can be seen from Figure 9c that the description ability of our descriptor is also reduced. Because the average curvature histogram is used in our descriptor, it improves the description ability while reducing the robustness to Gaussian noise.

#### 4.1.4. Robustness to Varying Mesh Resolution

**Figure 10:**
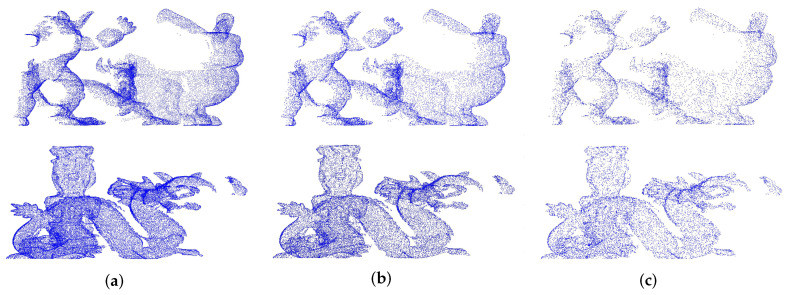
Examples of scenes with different mesh resolution. (**a**) 75%. (**b**) 50%. (**c**) 25%.

In order to evaluate the robustness of the descriptor to varying mesh resolution, 25%, 50%, and 75% downsampling are used, respectively, in the scene point cloud. Then, the feature descriptors based on the key points are calculated in the scenes. The feature descriptors of the model point cloud without noise are also calculated. The key point matching experiment are made for generating the P−R curve, and our descriptor would be contrasted with FPFH, RoPS, SHOT, and SpinImage through the experimental results. Here, with different mesh resolution, two examples of the scene point clouds are shown in Figure 10.

Here, the steps of the experiments are approximately identical to those of the previous section, except that the scenes are downsampled instead of adding noise.

**Figure 11:**
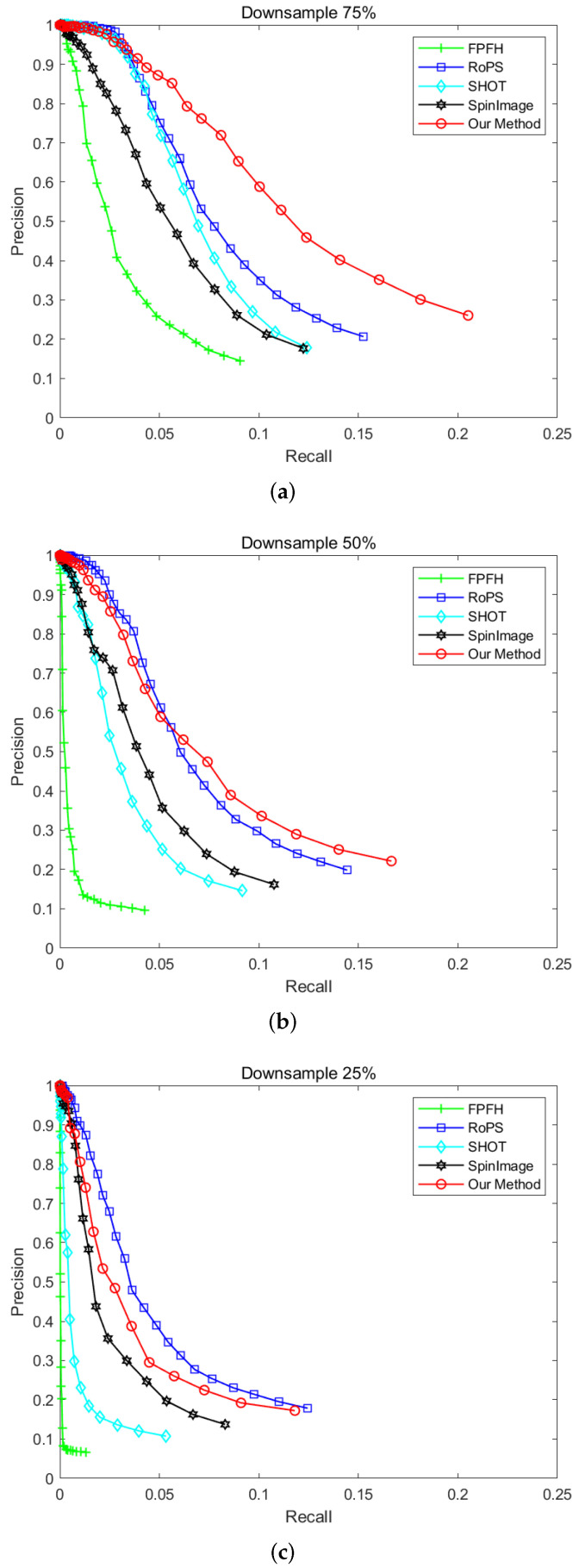
P-R curves in different mesh resolution. (**a**) Downsampling 75%. (**b**) Downsampling 50%. (**c**) Downsampling 25%.

The P−R curve in Figure 11 shows the performance of these different descriptors with different mesh resolution. It can be seen that our descriptor is more robust than other descriptors under different mesh resolution, and RoPS has the second best performance. As the proposed descriptor extracts the geometric attributes features of the points, such as the normals and curvatures, even if there are low mesh resolution, occlusion and incomplete regions in the scene point cloud, the description ability of our descriptor can be guaranteed. Although our descriptor does not perform well when the point clouds are downsampled to 25%, it is rare for this degree of mesh resolution in actual work. Moreover, our feature descriptor performs well when the mesh resolution downsampling to 75% and 50%. Therefore, our feature descriptor is robust to varying mesh resolution.

#### 4.1.5. Key Point Matching Based on Descriptors with Single Model

**Figure 12:**
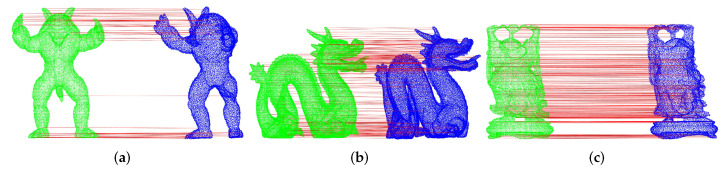
Examples of matching results between models and Gaussian noise models. (**a**) Corresponding key point pairs in Armadillo. (**b**) Corresponding key point pairs in Asian Dragon. (**c**) Corresponding key point pairs in Happy Buddha.

In this experiment, we use six point cloud models from the Stanford University dataset as the model point cloud. For the scene point cloud, the Gaussian noise (σ = 0.1 r) is added into each model, and then these point clouds are rotated and translated to a new position, so we can regard them as the scene point cloud. Moreover, the model point cloud without noise is still at the initial position. Now the experiment is to make the pairwise registration between the model point cloud and the scene point cloud. After the extraction of feature descriptors and the feature matching by NNDR, the correspondences have been constructed between the model point cloud and the scene point cloud. Figure 12 shows the examples about the results of the key point matching between the model point cloud (in green) and the scene point cloud (in blue). The red lines are used for connecting the corresponding key points.

In general, the more parallel red lines there are, the more correctly matched corresponding key point pairs there are. If a red line is not parallel to most other red lines, it represents the wrong matched corresponding key point pairs.

According to the results of the corresponding key point pairs, the SVD method is used for calculating the rotation matrix $R_{d}$ and the translation matrix $T_{d}$. The wrong matched corresponding key point pairs will cause errors to the rotation and translation matrix. Therefore, an effective feature descriptor can obtain more correctly matched corresponding key point pairs. For the scene point cloud at the above paragraph, and the real rotation matrix is defined as $R_{gt}$, and the translation matrix is defined as $T_{gt}$. If the error between $R_{d}$ and $R_{gt}$ is small and the error between $T_{d}$ and $T_{gt}$ is also small, it means there are many correctly matched corresponding key point pairs, also it can reflect that the descriptor have a good performance in pairwise registration. The rotation error $\theta_{r}$ and the translation error $\theta_{t}$ can be defined as follows:

(23) $$\theta_{r}=\arccos\left(\frac{trace(R_{d}R_{gt}^{-1}-1)}{2}\right)*\frac{180}{\pi }$$

(24) $$\theta_{t}=\frac{∥T_{d}-T_{gt}∥}{d_{r}}$$

Here, trace is the sum of the diagonal elements of the matrix and $d_{r}$ is set as 0.5 r.

Base on different descriptors, Table 1 shows the error of the rotation and translation after feature matching. The error of the rotation and translation calculated by the proposed descriptor is smaller than that of other descriptors. Thus, it can further prove that the description ability of our descriptor MSHD is better than other descriptors, and it can also reflect the robustness and effectiveness about our descriptor.

### 4.2. Matching Algorithm for Key Points between Model and Multi-Object Scene

**Figure 13:**
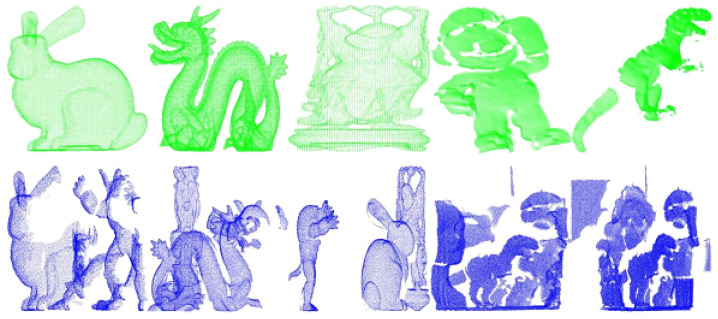
Point clouds that were selected in the experiment.

As the real point cloud data are usually collected by the laser scanner, it is inevitable that there will be occlusion, incomplete regions, etc. in the collected point cloud with multiple objects. As shown in Figure 13, for reflecting the characteristics of these real data, three models in Random View are selected: the Bunny, the Dragon, and the Happy Buddha (in green). Moreover, three scene point clouds containing these models are also selected (in blue). Furthermore, two models in Space Time dataset [5] are selected: the Mario and the Rex (in green). Two scene point clouds containing these models are selected (in blue). Therefore, there are five models and five scenes totally for the experiment. It can be seen that there are many occlusion, truncation, incomplete regions, and other problems in the each scene point cloud, while the model point cloud from Space Time dataset are single-view point cloud. These selected point clouds can restore the characteristics of real data, such as the multi-objects scenes and some single-view real data, which can help us to evaluate the effectiveness of our key point matching algorithm in this paper.

In this experiment, based on our descriptor, the key point matching algorithm is used for obtaining the corresponding key point pairs between the models and scenes, and then the rotation and translation matrix is calculated for 3D surface matching. Therefore, the models can be matched into the scene point cloud. The experimental results of our key point algorithm are compared with the commonly used NN and NNDR. Here, according to the principle, $\tau_{th}$ is set as 0.5 in NNDR, based on which the best performance can be got. All experiments are performed on the five model point clouds and the five scene point clouds that have been mentioned above.

**Figure 14:**
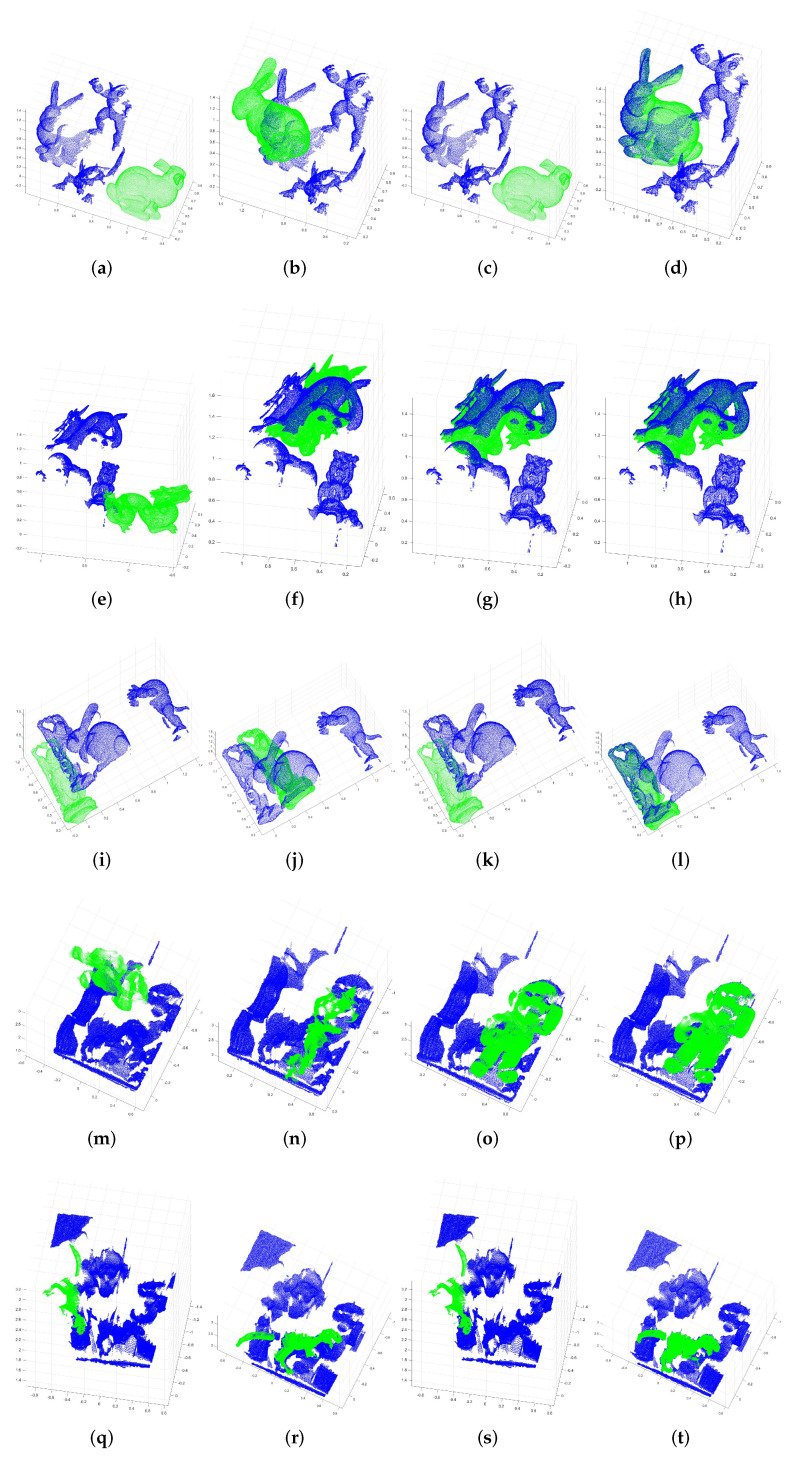
Surface matching results of three methods tested on the selected datasets. The models (in green) and the scenes (in blue) to be matched (**a**,**e**,**i**,**m**,**q**). NN results (**b**,**f**,**j**,**n**,**r**), NNDR results (**c**,**g**,**k**,**o**,**s**), and our method results (**d**,**h**,**l**,**p**,**t**).

The results of 3D surface matching are shown in Figure 14. It can be seen from the results of NN and NNDR, the model and the scene do not match well. Because the NN directly matches the most similar key point, and NNDR only considers the top-two similar key points in scenes. Many wrong matched corresponding key point pairs are obtained due to the limitation of these two algorithms, leading to the errors of transformation matrix which is calculated based on all the correspondences between the model and the scene point cloud, so the results of 3D surface matching are unsatisfied. Moreover, due to its strict conditions and the limitation of only considering the top-two similar points, in some situations, NNDR can not get enough or even any matched corresponding key point pairs. Less than three groups of corresponding point pairs will lead the transformation matrix can not be calculated, and the position of models also can not be transformed. In contrast, the 3D surface matching results of our key point matching algorithm are much better, which means there are much more correctly matched corresponding key point pairs.

Furthermore, it also can be seen from Table 2 that the error of our method is much smaller than NN. The word “matched” in the table means the number of the corresponding key point pairs. Analyzing from the results that combining with the errors of $\theta_{r}$ and $\theta_{t}$, based on our method, the number of correctly matched corresponding key point pairs is greater than that of NN and NNDR. NNDR cannot even get any matched corresponding key points pair in the data of Bunny, Happy Buddha, and Rex. Therefore, our method is more robust and effective in processing the data with occlusion, truncation, and incomplete regions.

### 4.3. Matching Algorithm for Real Data

**Figure 15:**
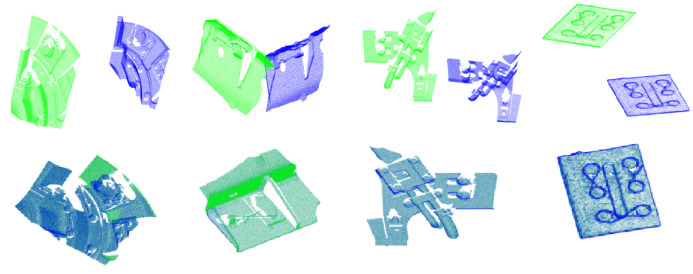
Results of 3D surface matching on real data. The up column is the initial position of these components. The down column is the results of 3D surface matching.

In this experiment, some real component point cloud data from the train bottom are used, and they are collected by the 3D laser scanning with a three-million pixel industrial camera, including the part of wheel hub, edge of base, tie rod, and bolts (Figure 15). All the real data have been preprocessed to improve the quality. The results of 3D surface matching are shown in Figure 15, and from Table 3 we can see that the error of our method is still much smaller than that of NN. NNDR is effective as ours, but more corresponding key point pairs can be obtained by our method, which is good for the last fine registration.

## 5. Conclusions

This paper introduces a 3D point cloud surface matching method, including a multi-statistics histogram descriptor that combines spatial distribution features and geometric attributes features, and a novel key point matching algorithm based on deep learning, which identifies more corresponding point pairs than the existing methods. Experimental results on Stanford dataset show that MSHD performs better than the baselines in the data with noise, occlusion, and incomplete regions. Meanwhile, MSHD has a strong robustness against noise and mesh resolution, and it also reflects a strong description ability. Our key point matching algorithm is evaluated on Stanford 3D dataset and four real component point clouds from the train bottom. From the results of the experiment about 3D surface matching, more corresponding key point pairs can be obtained. Combined with the results of errors in the rotation and translation matrix, it has been confirmed that the error of our methods is much smaller, and more number of precisely matched corresponding key point pairs can be captured, resulting in enhanced recognition and registration.

## Figures and Tables

**Table 1:** Errors of Stanford 3D models.

Model	Error	FPFH	RoPS	RoPS	SI	Ours
Armadillo	$\theta_{r}$	5.459	0.209	0.835	1.439	0.039
	$\theta_{t}$	0.933	0.011	0.548	0.337	0.056
Bunny	$\theta_{r}$	2.345	0.372	0.308	0.912	0.106
	$\theta_{t}$	0.670	0.013	0.147	0.156	0.003
Dragon	$\theta_{r}$	0.308	0.142	0.328	1.059	0.003
	$\theta_{t}$	0.221	0.004	0.079	0.105	0.053
Happy Buddha	$\theta_{r}$	3.301	0.095	0.061	1.575	0.017
	$\theta_{t}$	1.639	0.010	0.006	0.217	0.073
Asian Dragon	$\theta_{r}$	3.063	0.076	1.065	0.815	0.925
	$\theta_{t}$	0.239	0.076	0.015	0.006	0.004
Thai Statue	$\theta_{r}$	4.024	1.239	1.220	1.408	0.772
	$\theta_{t}$	0.237	0.014	0.039	0.012	0.006

**Table 2:** Errors of three key point matching algorithms on models and scenes.

Model	Error	NN	NNDR	Ours
Bunny	$\theta_{r}$	73.748		0.3858
	$\theta_{t}$	0.857	None	0.0534
	matched	42		10
Dragon	$\theta_{r}$	14.497	0	0
	$\theta_{t}$	5.548	4.0426$\;\times \;10^{-7}$	2.7387$\;\times \;10^{-7}$
	matched	318	6	8
Happy Buddha	$\theta_{r}$	18.082		1.1706
	$\theta_{t}$	7.643	None	0.0806
	matched	418		8
Mario	$\theta_{r}$	35.933	0.0115	0.3260
	$\theta_{t}$	39.396	3.0640$\;\times \;10^{-7}$	0.1596
	matched	28	3	7
Rex	$\theta_{r}$	112.570		0.0115
	$\theta_{t}$	33.651	None	2.4963$\;\times \;10^{-7}$
	matched	100		4

**Table 3:** Errors of the real data.

Model	Error	NN	NNDR	Ours
Wheel hub	$\theta_{r}$	2.233	0	0
	$\theta_{t}$	1.818	8.555$\;\times \;10^{-15}$	1.711$\;\times \;10^{-14}$
	matched	512	9	21
Edge of base	$\theta_{r}$	3.625	0	0
	$\theta_{t}$	4.806	1.711$\;\times \;10^{-14}$	1.711$\;\times \;10^{-14}$
	matched	512	8	12
Tie rod	$\theta_{r}$	1.252	0	1.1706
	$\theta_{t}$	0.995	2.851$\;\times \;10^{-15}$	5.704$\;\times \;10^{-15}$
	matched	512	10	15
Bolts	$\theta_{r}$	0.534	0	0
	$\theta_{t}$	0.511	1.083$\;\times \;10^{-13}$	5.703$\;\times \;10^{-15}$
	matched	512	3	98

## Data Availability

Not applicable.
